# Definitions and Prevalence of Multimorbidity in Large Database Studies: A Scoping Review

**DOI:** 10.3390/ijerph18041673

**Published:** 2021-02-09

**Authors:** Ying Pin Chua, Ying Xie, Poay Sian Sabrina Lee, Eng Sing Lee

**Affiliations:** 1 Lee Kong Chian School of Medicine, Nanyang Technological University, Singapore 639798, Singapore; ychua030@e.ntu.edu.sg; 2 Clinical Research Unit, National Healthcare Group Polyclinics, Singapore 138543, Singapore; xie_ying@nhgp.com.sg (Y.X.); sabrina_ps_lee@nhgp.com.sg (P.S.S.L.)

**Keywords:** multimorbidity, prevalence, definition, large database

## Abstract

Background: Multimorbidity presents a key challenge to healthcare systems globally. However, heterogeneity in the definition of multimorbidity and design of epidemiological studies results in difficulty in comparing multimorbidity studies. This scoping review aimed to describe multimorbidity prevalence in studies using large datasets and report the differences in multimorbidity definition and study design. Methods: We conducted a systematic search of MEDLINE, EMBASE, and CINAHL databases to identify large epidemiological studies on multimorbidity. We used the Preferred Reporting Items for Systematic Reviews and Meta-analysis Extension for Scoping Reviews (PRISMA-ScR) protocol for reporting the results. Results: Twenty articles were identified. We found two key definitions of multimorbidity: at least two (MM2+) or at least three (MM3+) chronic conditions. The prevalence of multimorbidity MM2+ ranged from 15.3% to 93.1%, and 11.8% to 89.7% in MM3+. The number of chronic conditions used by the articles ranged from 15 to 147, which were organized into 21 body system categories. There were seventeen cross-sectional studies and three retrospective cohort studies, and four diagnosis coding systems were used. Conclusions: We found a wide range in reported prevalence, definition, and conduct of multimorbidity studies. Obtaining consensus in these areas will facilitate better understanding of the magnitude and epidemiology of multimorbidity.

## 1. Introduction

Multimorbidity, the presence of multiple chronic conditions in an individual [1], challenges the current healthcare system [2]. Individuals with multimorbidity tend to have more complex healthcare needs, and effective management of their multiple chronic conditions is essential [3]. As the proportion of individuals with multimorbidity increases due to the aging population in most developed countries, issues threatening patient safety such as poor coordination of hospital processes, continuity of care, and polypharmacy have also become more prevalent [4,5].

With the exception of relatively uncommon conditions, the majority of visits for the management of chronic conditions and any co-existing conditions are made to primary care physicians, not specialists. Additionally, specialists play a greater role in managing specific conditions of their expertise, but not the other comorbid conditions that the patient may have [6]. Hence, our review chose to focus on the general and primary care populations.

The basic operational definition of multimorbidity includes the following parameters: the minimum number of chronic conditions to determine the presence of multimorbidity and the list of chronic conditions considered [7]. Acute conditions are not included in the definition of multimorbidity as these usually do not result in significant, long-lasting impact on patients’ lives [8]. Factors considered in the conduct of a multimorbidity prevalence study include the choice of study population, data sources, and diagnosis algorithm used. Diagnosis algorithms are used to determine the presence of a chronic condition in an individual, usually involving a combination of diagnosis codes, prescription data, or chronic disease databases [9]. Multimorbidity studies using large datasets require diagnosis algorithms to reliably pick up indicator chronic conditions in their study population. Different diagnosis algorithms used by different studies affect the pick-up rate of chronic diseases and subsequently the reported prevalence rates.

It is difficult to quantify the extent of the burden of multimorbidity, as the design of epidemiological multimorbidity studies varies greatly, and no consensus on the operational definition of multimorbidity exists [10]. A systematic review reported that prevalence was severely underestimated if studies used a list of fewer than 12 chronic conditions, while less variation existed in studies using more than 12 conditions [11]. The lack of consensus in the aforementioned areas resulted in a wide range of multimorbidity prevalence estimates and difficulty in comparing the prevalence among multimorbidity studies [10].

Larger samples are able to provide a more precise estimate of multimorbidity [12]. We defined large database studies as multimorbidity studies that used a study population greater than 500,000. This number was chosen to reflect a large dataset, as datasets of this size would likely have to store their data electronically. They are also more likely to be professionally governed and updated by a specialized data management team [13]. Compared to smaller databases, standards of governance are generally required to ensure diagnoses are reliably coded in large datasets.

Some challenges in using larger databases include the accuracy of the coding system used, and the reliability of coding as hospital records may be coded by non-physicians and may not reflect the actual codes from the physicians’ perspectives [14,15]. This is confounded by the impracticability to go through individual patient notes or obtain a direct account of the patient’s conditions. It has been suggested that patient record review is the best way to collect information about multimorbidity prevalence, as it is not reliant on coding and data entry but rather gathers data from the entire patient record [15,16]. However, this is not feasible in large database studies.

Therefore, a scoping review was conducted on multimorbidity studies using large datasets. The main objective was to describe the range of prevalence of multimorbidity reported by these studies. The secondary objectives of the review were to identify and report the differences in the definitions of multimorbidity and the conduct of these studies.

## 2. Materials and Methods

This scoping review was reported using Preferred Reporting Items for Systematic Reviews and Meta-analysis Extension for Scoping Reviews (PRISMA-ScR) protocol [17].

Articles were included if they were (a) written in English, (b) involved human participants, (c) had a study population greater than 500,000, (d) included the primary healthcare or general population, and (e) used electronic databases. Articles were excluded if they (a) focused only on comorbidity, (b) used patient-reported data, (c) studied only inpatients, (d) included acute conditions in the list of conditions, (e) used less than 12 conditions to define multimorbidity, or (f) were qualitative, interventional studies, reviews, editorials, systematic reviews, or meta-analyses.

The bibliographic databases of MEDLINE, EMBASE, and CINAHL were searched for all records from the inception date to 8 March 2020 to identify potentially relevant records. The search strategies were drafted and refined through team discussion amongst the authors. The final search strategies for the three databases can be found in Supplementary Table S1. The final search results were exported into EndNote, and duplicates were removed. The electronic database search was supplemented by hand searches of the references listed in the included articles and from Google Scholar.

The articles were screened by two independent reviewers (YPC and ESL) using Covidence. The reviewers sequentially evaluated the titles, abstracts, and then full text of all publications identified by our searches for potentially relevant articles. The reviewers resolved disagreements on article selection and data extraction by consensus and discussion with another reviewer (YX) if needed.

A data-extraction form was jointly developed by two independent reviewers (YPC and ZSC) to determine the type of information to extract. The form captured the relevant information on multimorbidity prevalence, definitions of multimorbidity used (e.g., minimum number of chronic conditions required, number of chronic conditions in the list and the list of chronic conditions), study settings (e.g., country of origin, year of publication and year of data extraction), and conduct of the studies (e.g., study design, population age and type, data sources, and diagnosis algorithms). The form also extracted information on the presence of any data governance or reliability standards of the electronic medical records used by the articles. The prevalence of multimorbidity was manually calculated from data provided in the articles if overall prevalence was not directly reported. In articles reporting prevalence estimates longitudinally over a period of time, we used prevalence estimates of the most recent data.

The two reviewers independently extracted the data, discussed the inputs, and updated the data-extraction form after resolving any disagreements through discussion. Consensus was reached by involving a third reviewer (ESL) for unresolved items.

The list of chronic conditions used by each article to define multimorbidity was compiled and organized into their respective categories based on the body systems. The organization of individual chronic condition into each category was conducted by two independent reviewers (YPC and ESL), and disagreements were resolved until consensus was reached via discussion with a third reviewer (YX). The organized conditions can be found in Supplementary Table S2.

## 3. Results

**Figure 1:**
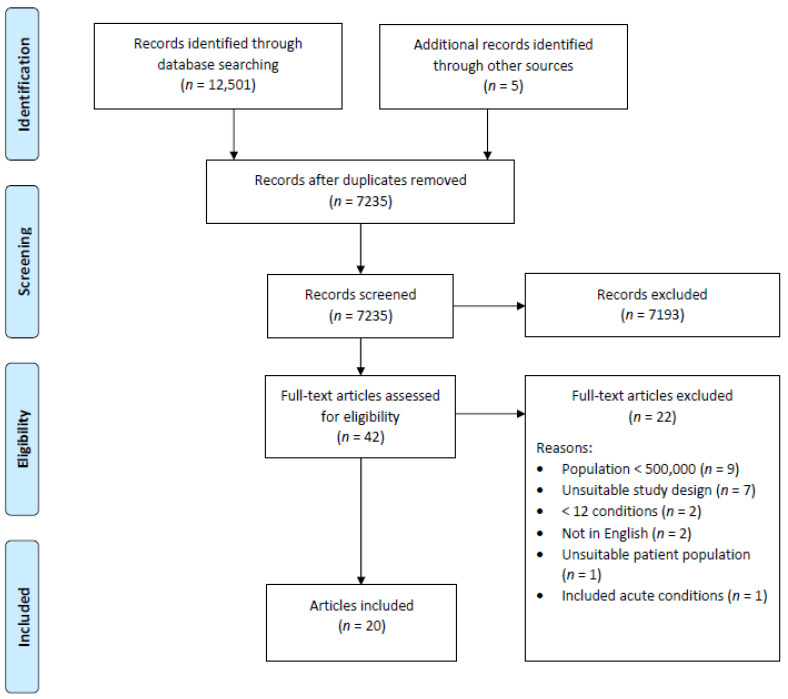
Preferred Reporting Items for Systematic Reviews and Meta-analysis (PRISMA) flow diagram.

A total of 7235 records were obtained from the searches, and 42 full text articles were assessed for eligibility. Of these, 22 were excluded for various reasons as shown in Figure 1. The remaining 20 articles were selected for this scoping review.

**Figure 2:**
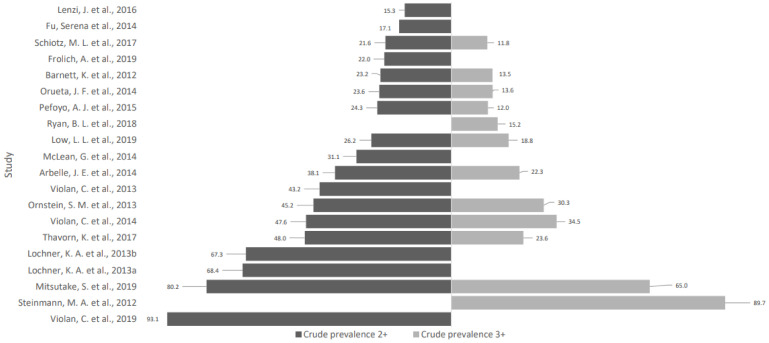
Crude prevalence of multimorbidity 2+ and 3+.

The study characteristics and definitions of multimorbidity used by the 20 articles are presented in Table 1. The chronic conditions used by each article were sorted into 21 body system categories, which are presented in Table 2. The prevalence of multimorbidity with at least two (MM2+) or three (MM3+) chronic conditions is presented in Figure 2. Two articles [18,19] did not provide sufficient data for the calculation of MM2+ prevalence, while eight articles [20,21,22,23,24,25,26,27] did not provide sufficient data for the calculation of MM3+ prevalence.

### 3.1. Study Characteristics

The majority of the included articles were from Europe (*n* = 9) [20,22,25,26,27,28,29,30,31], followed by North America (*n* = 7) [18,19,23,24,32,33,34] and Asia (*n* = 4) [21,35,36,37]. Seventeen of them were cross-sectional studies, while three articles were retrospective cohort studies [19,33,34].

There was significant variation in the age ranges of the study populations. Seven articles had no limits on age [21,23,24,28,29,35,37], seven articles included adults only [20,22,25,27,30,31,32], three articles included the elderly population only [19,26,36], and three articles included the whole population and only excluded people above the age of 105 [18,33,34]. The target population for majority of the articles was the general population (*n* = 9) [18,21,22,29,30,33,34,36,37], followed by articles that targeted the primary care populations (*n* = 8) [20,25,26,27,28,31,32,35] and insurance populations (*n* = 2) [23,24]. One article used a nationwide sample of community-dwelling elderly veterans receiving care in the United States’ Department of Veterans Affairs healthcare system [19].

### 3.2. Prevalence of Multimorbidity

The reported prevalence of multimorbidity was varied across the 20 articles. For MM2+ prevalence, 18 articles reported a prevalence ranging from 15.3% to 93.1%. For MM3+ prevalence, 12 articles reported a prevalence ranging from 11.8% to 89.7%. Nineteen articles reported crude prevalence rates, while one article [18] provided a standardized prevalence rate that was standardized against the 1991 Canadian population.

### 3.3. Definition of Multimorbidity

Most of the articles (85%, *n* = 17) used two chronic conditions and above to define the presence of multimorbidity. One article used at least three chronic conditions [18], one article used both two and three chronic conditions with a greater emphasis on three chronic conditions [36], while one article did not specify the number of chronic conditions [19].

The number of chronic conditions used in defining multimorbidity varied greatly from 15 to 147. Six out of 21 categories were present in the list of chronic conditions reported by all 20 articles. These six categories were “Cardiovascular”, “Endocrine”, “Mental health”, “Musculoskeletal”, “Neurology”, and “Respiratory”. The presence of the other categories was more diverse, with nine categories present in less than half (*n* = 10) of the articles. “Genetic conditions” and “Others” were two categories that were only present in two articles [29,31].

### 3.4. Diagnosis Codes and Algorithms Used

Eleven articles used diagnosis codes solely to denote the presence of chronic conditions. Nine articles used various additional means in their diagnosis algorithms for each condition [18,20,22,25,26,28,29,30,37], where prescription data as an additional means was used by all of them. The coding systems used were varied. A majority of studies used ICD-9-CM [18,19,21,22,23,24,29,32,33,34] or ICD-10-CM codes [18,20,26,27,30,33,34,35,36]; other codes used included Read2 [25] or ICPC-2 [31].

Out of the 20 included articles, only two of them described data governance or reliability standards of the electronic databases used. Arbelle et al. mentioned that the registries in their study were updated daily and automatically using strict algorithms. The algorithms drew data from numerous sources including physicians’ diagnoses, prescription information, data acquired from hospital discharge codes, and billing information from providers. The hospital discharge record database used in the study by Lenzi et al. underwent data quality control by the regional authority before being sent to the Ministry of Health.

## 4. Discussion

In this scoping review, 20 articles studying multimorbidity using large datasets were identified. There was significant variation in the reported prevalence and definitions of multimorbidity as well as in the conduct of the studies. This finding was aligned with existing literature, which suggested that reported multimorbidity prevalence is still highly varied due to inconsistent definitions of multimorbidity [11] in both large and small studies [38].

### 4.1. Definitions of Multimorbidity

Most articles used two chronic conditions and above (MM2+) to define the presence of multimorbidity. Only five articles provided the rationale for doing so: two were based on previous systematic reviews [22,28], and three were based on government regulations [23,24,37]. The remaining 15 articles, including those that used three chronic conditions and above (MM3+), did not provide any rationale for their choices. Lenzi et al. mentioned that MM3+ may be more useful in an older study population, but the more general definition of MM2+ was better applied to the general population [22]. The current literature suggested that the majority of the authors supported the use of MM2+ as the minimum number of chronic conditions to determine the presence of multimorbidity.

### 4.2. List of Conditions Used

The lists of chronic conditions used by the 20 articles ranged between 15 and 147 conditions. Most articles clustered around 15 to 30 conditions, which was partly due to the exclusion of articles with fewer than 12 conditions as recommended by Fortin et al. [11].

The following six categories of conditions were included in all 20 articles: cardiovascular, endocrine, mental health, musculoskeletal, neurology, and respiratory. This suggests greater relevance of these categories in the primary care and general population, which is possibly due to prevalence of conditions, such as acute myocardial infarction (cardiovascular), diabetes mellitus (endocrine), or greater impact on patients’ lives, such as requiring lifelong medications. To derive the lists of conditions, most articles based their lists on previous studies, which was followed by clinical relevance of the conditions. Less commonly, government guidelines, indexes, or systematic reviews were used.

### 4.3. Inclusion of Mental Health Conditions

All articles included mental health conditions, particularly depression, demonstrating that mental health conditions are significant health problems to consider in multimorbidity. Seven articles [20,22,25,28,30,35,37] grouped chronic conditions into overarching categories of physical and mental health conditions, of which four articles [25,28,30,35] separately analyzed the prevalence of physical–mental, purely physical, or mental health multimorbidity.

While specialists may better provide care for patients with one dominant disease or closely related comorbidities, the management of physical–mental multimorbidity requires holistic care and delicate balance [39]. For example, drugs prescribed for a physical condition may adversely affect mood, while synergistic treatment strategies can improve outcomes in patients with physical–mental multimorbidity [40]. Generalists may integrate the patient’s clinical problems, review medications, and assess the patient holistically [41], and a generalist primary care system is best equipped to manage physical–mental multimorbidity [28].

### 4.4. Diagnosis Coding Systems and Algorithms Used

Four coding systems were used by the 20 articles: ICD-9-CM, ICD-10-CM, Read2, and ICPC-2. ICD-9-CM and ICD-10-CM were most commonly used by the 20 articles. However, the code accuracy, defined as the extent to which the ICD nosologic code reflects the underlying patient’s disease, is usually low [42]. ICPC-2, designed specifically for the primary care setting, may inaccurately capture conditions less commonly seen in primary care. For all four coding systems, the use of synonyms, acronyms, and abbreviations in medical terminology results in differing codes selected [42], and accurate training is required to reduce coding errors [43]. Hence, coding accuracy and specificity of electronic health records differ amongst the 20 articles.

A lack of consensus on coding systems and diagnosis algorithms results in difficulty comparing among different multimorbidity studies. In studies using different coding systems, imperfect mapping of individual conditions onto a common coding system affects the accuracy of comparison studies. Differences in diagnosis algorithms also affect prevalence estimates of multimorbidity, as conditions may be under-reported if only diagnosis codes are used. Nearly half of the articles (*n* = 9) in this scoping review used prescription data as an additional means of diagnosing chronic conditions. Future studies may consider including prescription data or other means of confirming diagnoses in their diagnosis algorithms as well as standardizing them for large dataset multimorbidity studies to more accurately estimate multimorbidity prevalence.

Large databases are reliant on accurate clinical coding. Apart from inherent limitations of the coding systems used, ambiguity in patient record documentation and lack of clinical experience of coders affect coding accuracy [44]. While patient record review has been suggested as the best method to derive multimorbidity prevalence as it is not reliant on coding and data entry [15,16], large studies lack access to individual patient notes or direct accounts of patients’ conditions. Errors in coding subsequently implicate the accuracy of research using data from large databases [14].

As such, data governance by a professional body is essential to ensure the reliability of large databases. However, data governance in healthcare is less mature compared to other industries, and no universal standard for healthcare data exists [45]. Most of the articles (*n* = 18) did not mention any governance standards of the databases used as well. Indicating the presence of data governance is recommended to increase the reliability of multimorbidity studies using large databases.

### 4.5. Limitations

Our scoping review has some limitations. A categorical approach to analyzing the chronic conditions rather than individual comparison was chosen for feasibility reasons. This made the identification of key chronic conditions difficult.

## 5. Conclusions

In conclusion, our scoping review found a wide range in prevalence of multimorbidity as reported in studies using a large dataset, from 15.3% to 93.1% in MM2+ and 11.8% to 89.7% in MM3+. This is due to differences in both the definitions of multimorbidity and the conduct of the multimorbidity studies.

Consensus is urgently needed to facilitate comparison across studies as well as ensure reproducibility. Additional research such as a qualitative study using the Delphi method [46] may be important to get consensus where gold standards are absent to create a pre-defined list of key chronic conditions that should be included in multimorbidity studies for large dataset studies.

Methods of diagnosing chronic conditions will also need to be standardized, harmonizing the current established coding systems and diagnosis algorithms. This is especially important as large datasets are reliant on multiple factors to ensure reliability, such as standards of governance of electronic medical records, accuracy of data coding, diagnosis codes, and algorithms used.

## Figures and Tables

**Table 1:** A comparison of study characteristics and definitions of multimorbidity used in the 20 articles.

Article Number	Author, Year of Publication	Study Design	Country	Population Age	Population Type	Type of Database	Number of Conditions for MM Definition	Number of Chronic Conditions in List	Definition of Chronic Condition	Additional Means to Diagnose Conditions
1	Arbelle et al., 2014 [37]	CS	Israel	≥0	General population	EMR	2+	40	No	a. Prescription data b. Hospital discharge codes c. Billing info
2	Barnett et al., 2012 [28]	CS	Scotland	≥0	Primary care	EMR	2+	40	Yes	Prescription data
3	Frolich et al., 2019 [20]	CS	Denmark	>16	a. Primary care b. Secondary care	EMR	2+	16	No	a. Prescription data b. Healthcare service utilisation
4	Fu et al., 2014 [21]	CS	Taiwan	≥0	General population	Insurance database	2+	15	No	No
5	Lenzi et al., 2016 [22]	CS	Italy	≥18	General population	EMR	2+	26	No	Prescription data
6	Lochner et al., 2013 [23]	CS	U.S.	≥0	Insurance population	Insurance database	2+	15	No	No
7	Lochner. et al., 2013 [24]	CS	U.S.	≥0	Insurance population	Insurance database	2+	15	No	No
8	Low et al., 2019 [35]	CS	Singapore	≥0	a. Primary care b. Tertiary care c. Community hospitals	Government administrative data	2+	48	No	No
9	McLean et al., 2014 [25]	CS	Scotland	≥25	Primary care	EMR	2+	40	No	Prescription data
10	Mitsutake et al., 2019 [36]	CS	Japan	≥75	General population	Insurance database	2+ & 3+	22	No	No
11	Ornstein et al., 2013 [32]	CS	U.S.	≥18	Primary care	EMR	2+	24	No	No
12	Orueta et al., 2014 [29]	CS	Spain	≥0	General population	EMR	2+	52	No	Prescription data
13	Pefoyo et al., 2015 [33]	RC	Canada	0–105	General population	Insurance database	2+	16	No	No
14	Ryan et al., 2018 [18]	CS	Canada	0–105	General population	EMR	3+	17	No	Prescription data
15	Schiotz et al., 2017 [30]	CS	Denmark	≥16	General population	EMR	2+	16	No	a. Prescription data b. Healthcare service utilisation
16	Steinmann et al., 2012 [19]	RC	U.S.	≥65	Special group: Veterans	Government administrative data	DNS	23	No	No
17	Thavorn et al., 2017 [34]	RC	Canada	0–105	General population	EMR	2+	16	No	No
18	Violan et al., 2019 [26]	CS	Spain	≥65–99	Primary care	EMR	2+	60	No	a. Prescription data b. Other clinical parameters
19	Violan et al., 2013 [27]	CS	Spain	≥15	Primary care	EMR	2+	27	No	No
20	Violan et al., 2014 [31]	CS	Spain	≥19	a. Primary care b. Urban population	EMR	2+	147	No	No

Note: CS—cross-sectional, RC—retrospective cohort, DNS—did not specify, EMR—electronic medical records.

**Table 2:** Categories of conditions.

Category	Number of Articles in the Category	Arbelle, J. E., 2014	Barnett, K., 2012	Frolich, A., 2019	Fu, Serena, 2014	Lenzi, J., 2016	Lochner, K. A., 2013	Lochner, K. A., 2013	Low, L.L., 2019	McLean, G., 2014	Mitsutake, S., 2019	Ornstein, S. M., 2013	Orueta, J. F., 2014	Pefoyo, A. J., 2015	Ryan, B. L., 2018	Schiotz, M. L., 2017	Steinmann, M. A., 2012	Thavorn, K., 2017	Violan, C., 2019	Violan, C., 2013	Violan, C., 2014
1. Cardiovascular	20	✓	✓	✓	✓	✓	✓	✓	✓	✓	✓	✓	✓	✓	✓	✓	✓	✓	✓	✓	✓
2. Endocrine	20	✓	✓	✓	✓	✓	✓	✓	✓	✓	✓	✓	✓	✓	✓	✓	✓	✓	✓	✓	✓
3. Mental health	20	✓	✓	✓	✓	✓	✓	✓	✓	✓	✓	✓	✓	✓	✓	✓	✓	✓	✓	✓	✓
4. Musculoskeletal	20	✓	✓	✓	✓	✓	✓	✓	✓	✓	✓	✓	✓	✓	✓	✓	✓	✓	✓	✓	✓
5. Neurology	20	✓	✓	✓	✓	✓	✓	✓	✓	✓	✓	✓	✓	✓	✓	✓	✓	✓	✓	✓	✓
6. Respiratory	20	✓	✓	✓	✓	✓	✓	✓	✓	✓	✓	✓	✓	✓	✓	✓	✓	✓	✓	✓	✓
7. Neoplasia	19	✓	✓	✓	✓	✓	✓	✓	✓	✓	✓		✓	✓	✓	✓	✓	✓	✓	✓	✓
8. Rheumatology	15	✓	✓		✓	✓			✓	✓	✓	✓	✓	✓			✓	✓	✓	✓	✓
9. Urology/renal	15	✓	✓		✓	✓			✓	✓	✓		✓	✓	✓		✓	✓	✓	✓	✓
10. Gastrointestinal	14	✓	✓	✓	✓	✓				✓	✓	✓	✓		✓		✓		✓	✓	✓
11. Vascular	11	✓	✓			✓			✓	✓		✓	✓				✓		✓	✓	✓
12. Hepatopancreaticobiliary	10	✓	✓			✓			✓	✓		✓	✓		✓				✓		✓
13. Infectious diseases (communicable)	9	✓	✓		✓	✓				✓			✓		✓				✓		✓
14. Ophthalmology	9	✓	✓		✓					✓	✓		✓						✓	✓	✓
15. Dermatology	8	✓	✓						✓	✓			✓						✓	✓	✓
16. Disability	7	✓	✓			✓			✓	✓			✓								✓
17. Ear Nose and Throat	6		✓							✓			✓						✓		✓
18. Hematology	6										✓		✓				✓		✓	✓	✓
19. Immunologic	5			✓									✓			✓			✓	✓	
20. Genetic	2													✓						✓	
21. Others	2												✓								✓

Note: ✓ represents the presence of conditions from the particular category in the article. Each category consists of conditions involving said body system; “Others” includes “subfertility/infertility”, “weakness/tiredness general” and “transplant status”.

## Supplementary Materials

The searching methodology and list of conditions sorted by category are available online at https://www.mdpi.com/1660-4601/18/4/1673/s1.
