# Supplementary material for: Definitions and Prevalence of Multimorbidity in Large Database Studies: A Scoping Review
Source: Int J Environ Res Public Health. 2021 Feb 9;18(4):1673. doi: 10.3390/ijerph18041673 (PMC7916224; doi:10.3390/ijerph18041673)
Supplement: Supplementary file 1 [file ijerph-18-01673-s001.pdf]

Supplementary Materials

Supplementary Table S1: Searching methodology

Date of search: 8/3/2020

Supplemental table S1a: Ovid MEDLINE (R): 1946 to 2020 March 08

#	Searches	Results
1	multimorbidity/ or multiple chronic conditions/	1081
2	(multimorbid* or multi-morbid* or comorbid* or co-morbid*).ab,ti,kw.	143825
3	((multiple or coexist* or co-exist* or concurrent* or simultaneous*) adj2 (disease* or illness* or diagnos* or condition* or morbid* or disorder*)).ab,ti,kw.	43585
4	1 or 2 or 3	183995
5	Electronic health records/	18600
6	((Electronic or computer* or administrative) adj2 (record* or data*)).ab,ti,kw.	76483
7	(Large database* or database*).ab,ti,kw.	341698
8	5 or 6 or 7	390501
9	Prevalence/	283624
10	(Prevalen* or epidemio*).ab,ti,kw.	913241
11	9 or 10	980075
12	4 and 8 and 11	4040
13	Limit 12 to (English language and humans)	3905

Supplemental table S1b: Embase: 1974 to 2020 March 08

#	Searches	Results
1	exp Multiple chronic conditions/	2801
2	(multimorbid* or multi-morbid* or comorbid* or co-morbid*).ab,ti,kw.	319120
3	((multiple or coexist* or co-exist* or concurrent* or simultaneous*) adj2 (disease* or illness* or diagnos* or condition* or morbid* or disorder*)).ab,ti,kw.	77586
4	1 or 2 or 3	388894
5	Electronic health record/ or electronic medical record/	68868
6	(Electronic or computer* or administrative*) adj2 (record* or data*).ab,ti,kw.	153076
7	(Large database* or database*).ab,ti,kw.	635643
8	5 or 6 or 7	741513
9	Prevalence/	694889
10	(Prevalen* or epidemio*).ab,ti,kw.	1465157
11	9 or 10	1596491
12	4 and 8 and 11	10464
13	limit 12 to (English language and humans)	9815
14	Limit 13 to (article or article in press)	4436

Supplemental table S1c: CINAHL: 1984 to 2020 March 08

#	Searches	Results
1	(MH “comorbidity”)	56629
2	multimorbid* or multi-morbid* or comorbid* or co-morbid*	97530
3	(multiple or coexist* or co-exist* or concurrent* or simultaneous*) N2 (disease* or illness* or diagnos* or condition* or morbid* or disorder*)	18589
4	1 or 2 or 3	113365
5	(MH “electronic health records”)	22313
6	(Electronic or computer* or administrative) N2 (record* or data*)	138566
7	Large database* or database*	168616
8	5 or 6 or 7	272365
9	(MH “prevalence”)	85505
10	Prevalen* or epidemio*	553492
11	9 or 10	553492
12	4 and 8 and 11	5300
13	Limit 13 to (English language and humans)	4160

Supplementary Table S2: Conditions sorted by category

Cardiovascular conditions	Dermatological conditions	Endocrinological conditions	ENT conditions	Gastrointestinal conditions	Genetic conditions	Urological/renal conditions	Haematological conditions	Hepatopancreaticobiliary conditions	Immunological conditions	Infectious diseases (communicable)	Mental health conditions	Musculoskeletal conditions	Neoplasia	Neurological conditions	Ophthalmological conditions	Respiratory conditions	Rheumatological conditions	Vascular conditions (different from haematological?)	Disability	Others
Acute myocardial infarction	Acne	Diabetes	Chronic sinusitis	Chronic constipation	Chromosomal abnormalities	Benign prostatic hypertrophy	Anaemia	Cholecystitis/cholelithiasis	Allergies	AIDS/HIV	Alcohol problems	Arthritis	Neoplasia/cancer	Alzheimer's disease and related dementia	Blindness and low vision	Asthma	Gout	Atherosclerosis	Congenital anomaly digestive system	Infertility/subfertility
Angina	Chronic ulcer of skin	Diabetes mellitus	Deafness	Chronic enteritis/ulcerative colitis	Chromosomal anomalies or Inherited metabolic disorders	Chronic kidney disease	Anaemia, other/unspecified	Chronic liver disease	Autoimmune disease	Chronic infectious disease	Affective psychosis	Acquired deformity of spine	Any malignancy including lymphoma and leukemia except malignant neoplasm of skin	Attention deficit disorder	Blindness	Bronchiectasis	Gout and other crystal arthropathies	Atherosclerosis/peripheral vascular disease	Congenital anomaly digestive system	Weakness/tiredness general
Arrhythmia	Dermatitis, seborrhoeic	Diabetes mellitus (T1 and T2)	Deafness, hearing impairmt	Colitis and related disease		Chronic kidney disease on dialysis/predialysis	Anaemia, Vit B12/folate deficiency	Chronic liver or pancreatic disease	Chronic allergies	Hepatitis	Alcohol use disorders	Arthritis (excluding rheumatoid arthritis)	Benign neoplasm nervous system	Cerebrovascular accident	Blindness, visual impairment	Chronic obstructive pulmonary disease	Hyperuricemia	Circulation disorders (other than varicose veins)	Congenital anomaly endocrine/metabolic	Transplant status
Atrial fibrillation	Dermatitis/atopic eczema	Diabetes, insulin dependent	Deafness, hearing loss	Combination category of gastroesophageal reflux disease, peptic ulcer disease, and dyspepsia (GERD/PUD)		Chronic renal insufficiency	Blood and blood-forming organ disease	Chronic pancreas, biliary tract and Gallbladder disease	Disorders of the immune system	HIV	Anorexia nervosa/bulimia	Back pain	Benign/unspecified neoplasm blood	Cerebrovascular disease	Cataract/other lens disease	Chronic obstructive pulmonary disease and asthma (COPD)	Inflammatory arthropathies	Peripheral arterial disease (PAD; including aortic aneurysms)	Congenital anomaly neurological	
Atrial fibrillation/flutter	Psoriasis	Diabetes, non-insulin dependent	Ear Nose and Throat disease	Constipation (treated)		Genital disease, other	Haematologic disorders (chronic)	Chronic pancreatic disease		HIV infection/AIDS	Anorexia/bulimia	Back syndrome with radiating pain	Cancer	Cluster headache	Cataract/glaucoma	Emphysema, chronic bronchitis, chronic obstructive pulmonary disease	Rheumatic disease	Peripheral vascular disease	Congenital anomaly NOS/multiple	
Bradycardias and conduction disease	Psoriasis or eczema	Dyslipidemia	Goitre	Digestive disease		Genitourinary disease	Hereditary haemolytic anaemia	Chronic viral hepatitis		Tuberculosis	Anxiety	Back syndrome without radiating pain	Cancer (breast, colorectal, lung, prostate)	Dementia	Cataracts	Other respiratory disease	Rheumatoid arthritis	Phlebitis/thrombophlebitis	Congenital anomaly, cardiovascular	
Cardiac arrhythmia	Skin disease, other	Endocrine/metabolic/nutritional disease, other	Hearing loss	Disease digestive system, other		Glomerulonephritis/nephrosis	Purpura/coagulation defects	Liver disease		Viral hepatitis	Anxiety disorder/anxiety state	Carpal tunnel syndrome	Cancer (without mets)	Dementia including Alzheimer's	Eye disorders	Respiratory disease	Rheumatoid arthritis and autoimmune and connective tissue diseases	Pulmonary embolism	Congenital anomaly, musculoskeletal	
Cardiac arrhythmia NOS	Skin diseases	High cholesterol	Hypertrophy tonsils/adenoids	Diverticular disease		Kidney transplant		Liver disease NOS			Anxiety, dissociative and somatoform disorders	Chronic back pain	Haematological neoplasms	Epilepsy	Glaucoma	Respiratory disease, other	Rheumatoid arthritis, other inflammatory polyarthropathies & systematic connective tissue disorders	Varicose veins	Developmental disorder	
Cardiac disease		Hypercholesterolemia	Presbyacusis	Diverticular disease of intestine		Nephritis		Moderate liver disease			Anxiety/neurotic/stress disorders	Degenerative joint disease	Hodgkin's disease/lymphoma	Epilepsy (currently treated)	Macular degeneration	Respiratory failure	Rheumatoid/seropositive arthritis	Varicose veins of leg	Intellectual disabilities	
Cardiac valve disease		Hyperlipidemia	Vertiginous syndrome	Duodenal ulcer		Nephrosis		Severe liver disease			Bipolar disorder	Dorsopathies	Leukemia	Haemorrhagic stroke	Other eye disease			Venous and lymphatic disease	Learning disability	
Cardiovascular disease		Hyperthyroid		Dyspepsia (treated)		Other genitourina					Chronic alcohol abuse	Hip fracture	Malignancies	Hyperkinetic disorder	Retinopathy				Major lower	

			ry diseases													extremity amputation
Chronic coronary syndrome	Hyperthyroidism/thyrotoxicosis	Gastroesophageal reflux	Pelvic inflammatory disease					Depression	Joint disease	Malignancy NOS	Insomnia					Mental retardation
Chronic heart disease, others	Hypothyroid	Haemorrhoids	Prostate disease					Depression and mood diseases	Low back pain	Malignancy other than prostate cancer	Ischemic stroke					Minor lower extremity amputation
Congestive heart failure	Hypothyroidism	Inflammatory bowel disease	Prostatic disorders					Depression or anxiety	Musculoskeletal disease, other	Malignant neoplasm bladder	Migraine					
Coronary artery bypass graft (CABG)	Hypothyroidism/myxoedema	Irritable bowel syndrome	Prostatic hypertrophy					Depressive disorder	Neck pain	Malignant neoplasm blood other	Migraine and facial pain syndrome					
Coronary disease	Hypthyroidism	Oesophagus, stomach and duodenum disease	Renal disease					Dysthymia	Neck syndrome	Malignant neoplasm breast female	Migraine or frequent headaches					
Coronary heart disease	Lipid disorder	Other digestive disease	Renal failure					Generalised anxiety disease	Osteoarthritis	Malignant neoplasm bronchus, lung	Multiple sclerosis					
Heart disease	Lipid disorders	Peptic ulcer	Urinary disease, other					Long term use of antidepressants	Osteoarthritis and arthritis not otherwise specified (herein called arthritis)	Malignant neoplasm cervix	Nervous system disorders					
Heart disease, other	Obesity	Peptic ulcer disease	Urinary incontinence					Major depression	Osteoarthritis and degenerative joint disease	Malignant neoplasm colon/rectum	Neuraesthenia, surmenage					
Heart failure	Other metabolic disease	Peptic ulcer other	Urologic disease					Mental disorders	Osteoarthritis of hip	Malignant neoplasm genital female other	Neurological disease, other					
Heart valve disease NOS	Overweight	Peptide ulcer						Mood disorder	Osteoarthritis of knee	Malignant neoplasm kidney	Neurological infection, other					
Heart/arterial murmur NOS	Thyroid disease							Neurotic, stress-related and somatoform diseases	Osteoarthritis, arthritis or rheumatism	Malignant neoplasm male genital, other	Other neurological diseases					
Hypertension	Thyroid disorders							Organic psychosis, other	Osteoarthritis, other	Malignant neoplasm nervous system	Other neurological disorders					
Hypertension, complicated	Thyroidal disease							Other mental disorders	Osteoarthritis/spine disorder	Malignant neoplasm of skin	Parkinson and parkinsonism					
Hypertension, uncomplicated								Other psych and behavioural diseases	Osteoporosis	Malignant neoplasm pancreas	Parkinsonism					
Ischaemic heart disease with angina								Other psychoactive substance misuse	Painful condition	Malignant neoplasm prostate	Parkinson's disease					
Ischaemic heart disease without angina								Personality disorder	Paralysis	Malignant neoplasm related to fertility	Parkinson's disease or syndrome					
Ischemic heart disease								Personality disorders	Paralysis or muscular dystrophy	Malignant neoplasm respiratory, other	Peripheral neuritis/neuropathy					

Myocardial infarction	Phobia/compulsive disorder	Shoulder syndrome	Malignant neoplasm stomach	Peripheral neuropathy
Other cardiovascular disease	Post-traumatic stress disorder	Spine fracture	Malignant neoplasm thyroid	Peripheral neuropathy, neuritis
Paroxysmal tachycardia	Psychological disorders, other	Tennis elbow	Malignant neoplasm, musculoskeletal	Sleep disorder
Percutaneous coronary intervention (PCI)	Psychosis NOS/other		Malignant neoplasm, urinary, other	Stroke
Postural hypotension	Schizophrenia		Malignant tumours	Stroke and transient ischemic attack
Pulmonary heart disease	Schizophrenia (and related non-organic psychosis)		Metastatic carcinoma	Stroke/cerebrovascular accident
Rheumatic fever/heart disease	Schizophrenia and delusional disease		Metastatic solid tumour	Transient cerebral ischaemia
Secondary hypertension	Schizophrenia and psychosis		Neoplasm endocrine other/uncertain	Trigeminal neuralgia
	Schizophrenia, affective psychosis or bipolar disorder		Neoplasm nervous system, unspecified	
	Somatisation disorder		Neoplasm of ear	
	Substance use disorders		Neoplasm of eye/adnexa	
			Neoplasm, cardiovascular	
			Prostate cancer	
